# Complex Abdominal Wall Hernias: Structured Use of Adjuvant Therapies

**DOI:** 10.3389/jaws.2025.14515

**Published:** 2025-09-01

**Authors:** Joana Marques‐Antunes, Egon Rodrigues, Marta Guimarães, Ana Marta Pereira

**Affiliations:** ^1^ Department of General Surgery, Hospital São Sebastião, Unidade Local de Saúde de Entre Douro e Vouga, (ULSEDV), Santa Maria da Feira, Portugal; ^2^ Unit for Multidisciplinary Research in Biomedicine (UMIB), Porto, Portugal; ^3^ School of Medicine and Biomedical Sciences (ICBAS), University of Porto, Porto, Portugal

**Keywords:** abdominal wall reconstruction, preoperative progressive pneumoperitoneum, intraoperative fascial traction, botulinum toxin type A, complex hernia

## Abstract

**Purpose:**

Repairing complex abdominal wall hernias is challenging, often requiring component separation techniques (CST) for tension-free closure. Adjuvant therapies, such as botulinum toxin type A (BTA), preoperative progressive pneumoperitoneum (PPP), and intraoperative fascial traction (IFT), may reduce the need for CST by improving abdominal wall compliance and reduce the complexity of the hernia. There is limited knowledge about the effects of their combined use. Our aim is to evaluate the rate of CST in abdominal wall reconstruction for complex midline hernias after adjuvant therapies.

**Methods:**

A cross-sectional study was conducted on patients who underwent surgery for correction of midline complex abdominal hernias between June 2020 and June 2024. Patients submitted to BTA, PPP, or/and IFT were included. Exclusion criteria were non-midline hernias, non eletive surgeries and less than 3 months of follow-up.

**Results:**

Among the 44 patients studied, 61.4% underwent abdominal wall reconstruction without requiring CST. Traditional predictors like component separation index and rectus/defect ratio were not associated with a higher rate of CST after adjuvant therapies. 45.5% of patients underwent a combination of adjuvant techniques (BTA + PPP or BTA + IFT). The early and late complication rates were 20.5% and 9.1%. A recurrence rate of 4.5% was reported after a median follow-up of 13 months.

**Conclusion:**

This study suggests that adjuvant therapies may influence the surgical approach to abdominal wall reconstruction. The synchronous application of adjuvant therapies, both preoperatively and intraoperatively, could enhance their effect and contribute to the use of less disruptive techniques.

## Introduction

Repair of complex abdominal wall hernias entails a significant challenge for surgeons. The abdominal wall dysfunction is not only due to the hernia itself but also to the intrinsic muscle impairment [1]. Chronic muscle retraction typically reduces abdominal cavity volume. The reconstruction of the linea alba resembles to reverse the lateral retraction and fibrosis of the musculature [2, 3]. Restore the anatomy and function of the abdominal wall through a tension-free, mesh-reinforced, midline fascial closure is the main goal of surgery.

Rives-Stoppa retrorectus repair allows reconstruction of the abdominal wall by placing the mesh in a vascularized space, which promotes incorporation on the tissue and avoids subcutaneous dissection [4]. In some cases, achieving primary and tension-free myofascial closure is impossible with this technique, and component separation techniques (CST), such as transversus abdominis release (TAR), are required [5]. CST are more challenging to perform and can lead to irreversible disruption of the abdominal wall anatomy, as well as an increased risk of perioperative complications [6]. Alternatively, adjuvant therapies, such as injection of botulinum toxin type A (BTA), progressive pneumoperitoneum (PPP) or intraoperative dynamic fascial traction (IFT) can be used to increase abdominal wall compliance, avoiding CST [7–9].

BTA induces temporary paralysis of the lateral abdominal muscles, leading to their elongation and thinning. Simultaneously, the abdominal shape becomes more ovoid [10]. On the other hand, PPP increases the volume of the abdominal cavity by stretching the abdominal wall muscles and facilitates the reduction of herniated contents through the pneumatic dissection of adhesions [11–13]. The combined effects of both techniques can facilitate effective hernia downsizing. While BTA induces relaxation of the anterolateral abdominal muscles, PPP gradually expands the abdominal cavity, creating a more conducive environment for tension-free closure of the abdominal wall. Additionally, the use of BTA reduces the duration required for PPP insufflation [14, 15]. However, certain patients with hernias with significant myofascial retraction may benefit more from the administration of BTA followed by IFT - a controlled and time-limited mechanism designed to stretch the anterior aponeurosis during surgery [9, 16].

Preoperative planning is the cornerstone to achieve successful surgical outcomes. Based on the preoperative computed tomography (CT) some criteria can predict if the closure of the fascia will require CST, such as defect width greater than 8 cm, a rectus/defect ratio (RDR) below 1.34 [17], or a component separation index (CSI) exceeding 0.146 [18]. Guidelines recommend that only surgeons skilled in advanced techniques should perform surgery on hernias that meet or surpass these criteria [19].

Although some studies have already addressed the effects of adjuvants on the abdominal wall [10, 20], it remains unclear whether their application, particularly when used simultaneously, impacts the surgical technique used for the repair of complex midline hernias.

Our aim is to evaluate the rate of CST in abdominal wall reconstruction for complex midline hernias following adjuvant therapies, either alone or in combination, and to propose a structured algorithm for their use.

## Methods

### Design and Setting

This was an cross-sectional study of patients operated by the Complex Abdominal Wall Unit of our centre from June 2020 to June 2024. Written informed consent was obtained from the individuals for the publication of any potentially identifiable images or data included in this article. Ethical approval was obtained from the local ethics committee (CES n° 31_2024). The data were extracted from the electronic medical records. The study was designed and reported following the STROBE (Strengthening the Reporting of Observational Studies in Epidemiology) statement to ensure transparency and completeness in reporting.

### Participants

Inclusion criteria were patients who underwent midline complex abdominal hernia repair for primary or incisional hernias that were assigned to adjuvant therapies such as BTA administration, PPP, or IFT. Exclusion criteria were non-midline hernias (lateral, parastomal, synchronous ventral and lateral or inguinal), non eletive surgeries, surgeries performed outside our hospital or operated on by individuals not belonging to the complex abdominal wall surgery unit. Patients with less than 3 months of follow-up were also excluded.

### Variables and Data Measurement

The primary outcome was the rate of CST surgeries with the use of adjuvants. The secondary outcome was the complication rate and recurrence. Complications were classified according to the Clavien-Dindo classification [21]. Recurrence was considered whenever confirmed by CT scan. The tertiary outcome was to find predictors for CST with the use of adjuvants. Hernia´s location and size were reported according to the EHS classification [22]. Defect width was measured. Loss of domain was defined as hernia/abdominal volume ratio greater than 20% [23]. Imaging predictors of abdominal wall closure such as RDR [17] and CSI [18] were calculate based on preoperative CT scans.

### Preoperative Planning

All patients followed a preoperative program that included respiratory physiotherapy, smoking cessation, glycemic control, and comorbidities optimization in a multidisciplinary collaboration with endocrinology, pulmonology, internal medicine, and other medical specialties.

Patients with obesity were also evaluated by a multidisciplinary obesity team, with access to all treatment options, including bariatric surgery when deemed appropriate. Whenever possible, a staged surgical approach was adopted, with bariatric surgery performed first to achieve a lower BMI before proceeding with abdominal wall reconstruction. A synchronous approach, involving simultaneous bariatric surgery and abdominal wall reconstruction, was reserved for cases where primary laparoscopic surgery was not feasible. If the patient was deemed ineligible for bariatric surgery, they were enrolled in a non-surgical weight‐loss program.

### Adjuvants Protocols

Selection of adjuvants was determined according to EHS hernia classification [22], CT scan volumetric analysis of the hernia and abdominal cavity, as well as the surgeon´s clinical judgment. Adjuvants were used either preoperatively or intraoperatively.

Adjuvant therapy with BTA was performed in all hernias with width ≥8 cm or lateral muscles thickness ≥ 1 cm. Hernias with loss of domain were also assigned to BTA. The administration was ultrasound-guided, 4–6 weeks before surgery, by an abdominal wall surgeon. All patients received 500 U of Dysport in two or three points on both sides of the abdominal wall.

**FIGURE 1:**
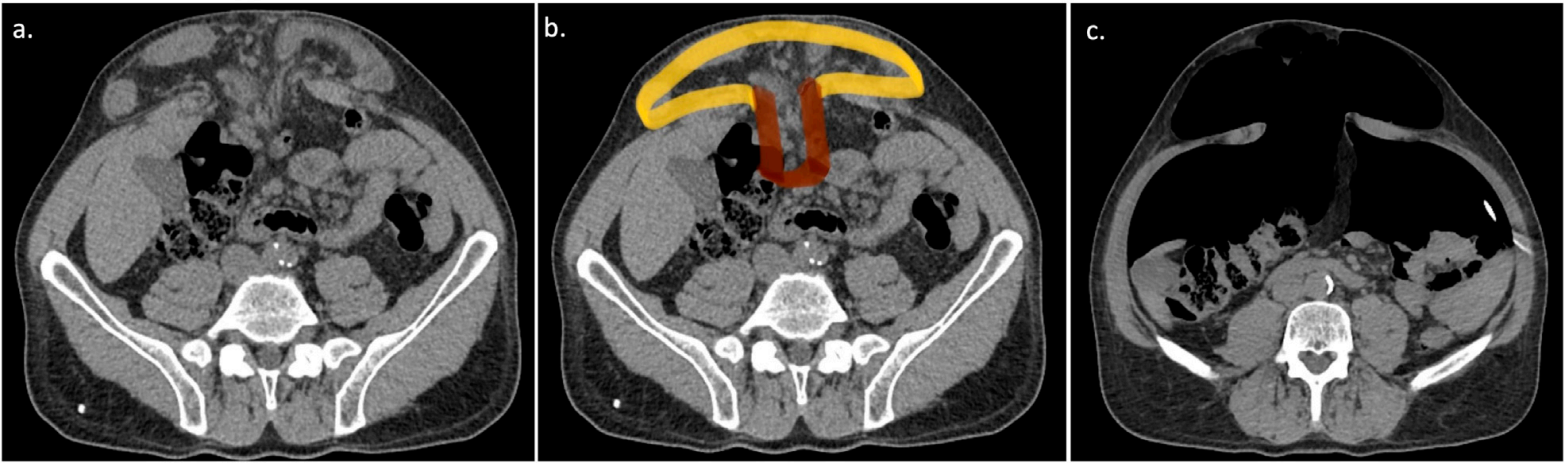
“Mushroom” pattern is characterized by a relatively small hernia defect in hernias with large volume and loss of domain. A CT scan of a complex midline hernia with “mushroom” pattern is shown before **(A,B)** and after the PPP **(C)**.

PPP was indicated for hernias with loss of domain and a simultaneously tight defect, characterized by what we termed the “mushroom” pattern (Figure 1). Patients were admitted 5–10 days before the surgery. A pigtail was inserted into the abdominal cavity. The insufflation was done daily with 500–1000 cc of air. The amount of air insufflated was calculated according to the herniary sac volume (HSV), employing the formula: Volume = 3x HSV +10% [24]. Venous thromboembolism prophylaxis was performed using elastic compression stockings, administration of prophylactic enoxaparin (40 mg/day), and walking for a minimum of 1–2 h each day.

**FIGURE 2:**
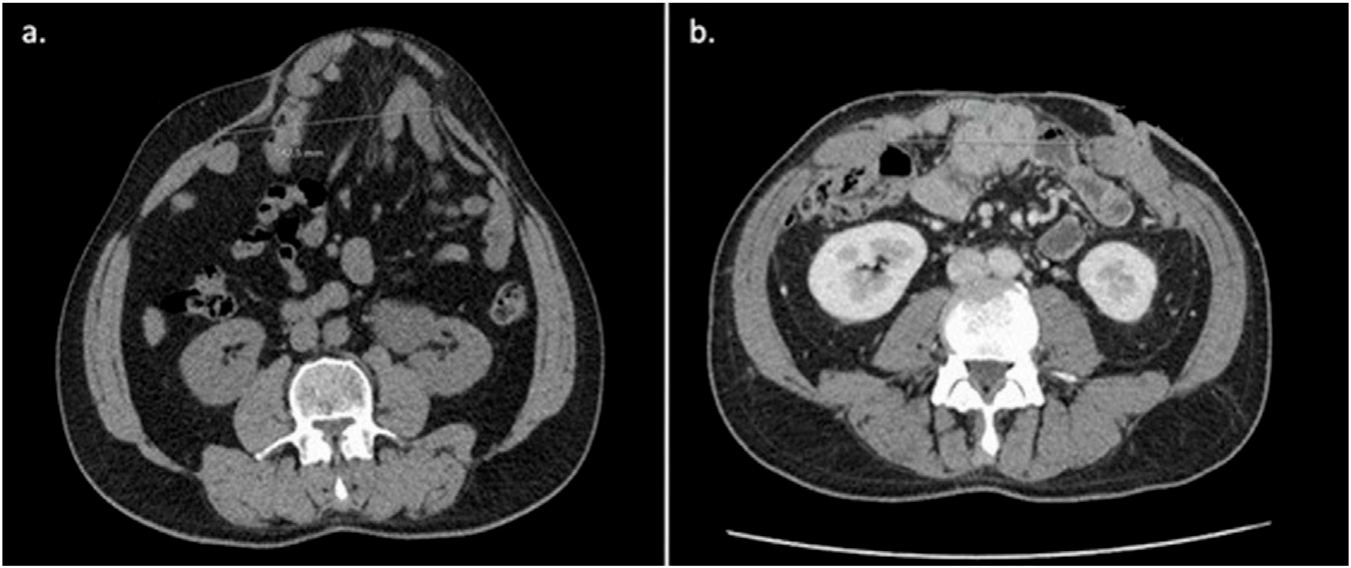
Examples of lateral muscle retraction on CT patients submitted to BTA and IFT. Lateral muscle retraction is indirectly estimated by observing the relationship between an increase in muscle thickness and a corresponding decrease in muscle length. The decrease in muscle length is typically associated with increased hernia width.

IFT was performed for W3 hernias with significant lateral muscle retraction, wherein closure of the abdominal wall was unachievable without a bilateral CST. The decision to use this adjuvant could be made intraoperatively or predicted preoperatively (Figure 2).

**FIGURE 3:**
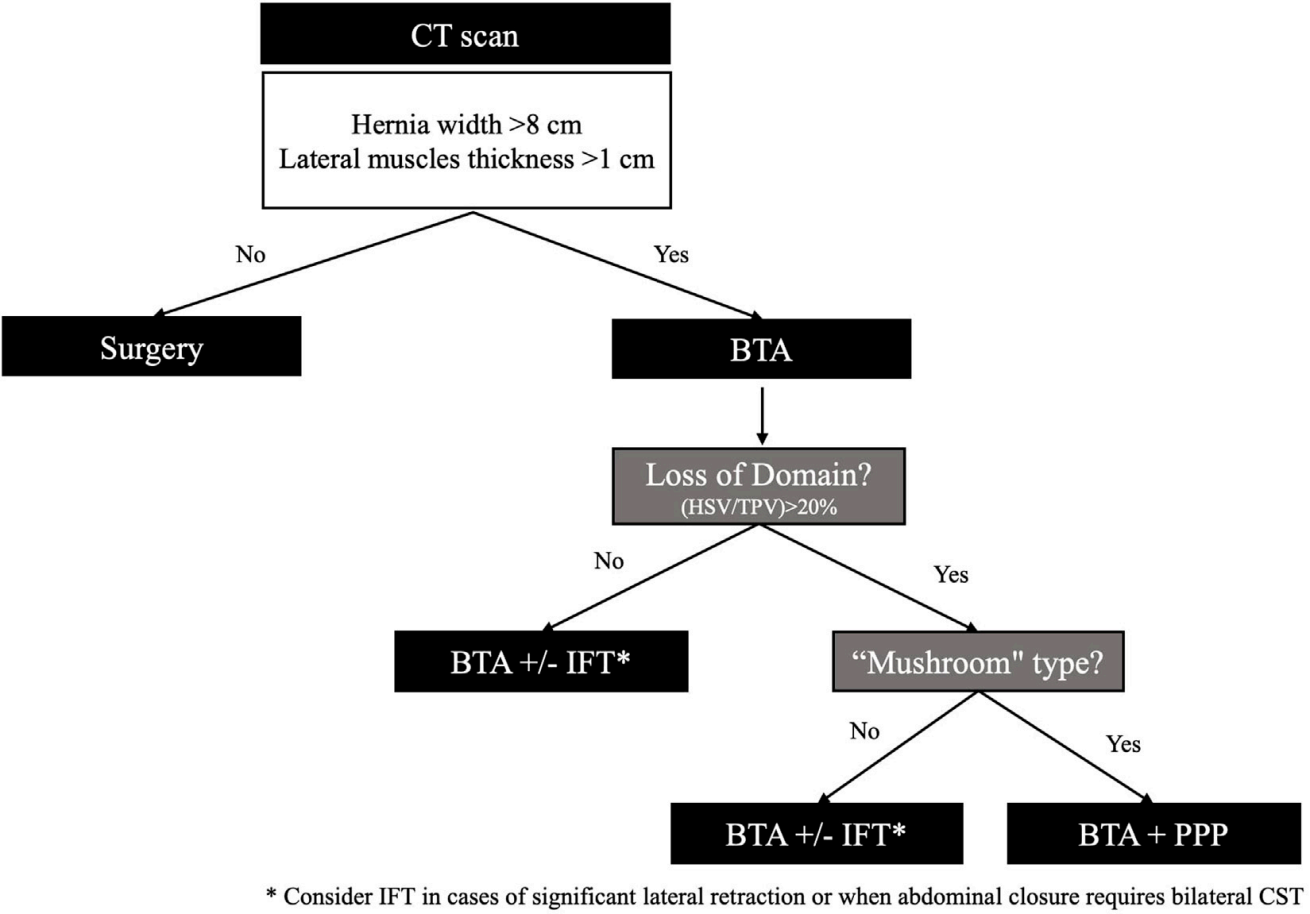
Algorithm for combined use of adjuvant therapies. BTA: botulinum toxin type A, PPP: preoperative progressive pneumoperitoneum; IFT intraoperative fascial traction; TPV: total peritoneal volume; HSV: hernia sac volume; CST: component separation techniques.

The strategy for combining adjuvants was grounded in their potential to synergistically enhance each other’s effects: adjuvants with muscle relaxation and elongation properties (BTA) were paired with those capable of inducing muscle stretching according to hernia characteristics (PPP or IFT) (Figure 3).

### Surgical Technique

We performed an open approach in all cases. The lateral dissection of the retrorectus space is carried out till the EIT Ambivium [25]. If the posterior rectus sheath could be closed without tension, we performed a Rives-Stoppa technique. Otherwise, the dissection progressed to a TAR. A macroporous, non-absorbable mesh was placed in the retromuscular plane covering the dissected area. Panniculectomy was performed if necessary, either through transverse or longitudinal incisions. In selected cases, anterior component separation was necessary when posterior component separation was not feasible due to prior violation of the retromuscular plane from previous surgeries, including those for hernia repair. Hybrid techniques, staged approaches, or placement of mesh in more than one plane could be necessary according to the local conditions. All the surgical repairs were performed by surgeons of the Complex Abdominal Wall Unit, formed by three surgeons.

### Postoperative Follow-Up

Patients were clinically reevaluated at 1 week, 6 months, and 2 years postoperatively. A CT scan was performed whenever there was suspicion of recurrence or complications.

### Data Analysis

Categorical variables were quantified as the number of cases and percentage (%) and continuous variables were reported as mean ± standard deviation, or median with interquartile range, as applicable. The statistical analysis was performed using the chi-squared test for categorical variables and the independent t-test or the Mann-Whitney U test for continuous variables, as applicable. Statistical significance was defined by p < 0.05. SPSS. Version 29.0.0.0 was used for statistical analysis.

## Results

A total of 58 patients underwent abdominal wall reconstruction after adjuvant therapy. Twelve (22.4%) were excluded due to hernia location (lateral, parastomal, synchronous ventral and lateral, inguinal), and two (3.4%) due to less than 3 months of follow-up.

Preoperative patient demographic and hernia characterization are presented in Table 1. The mean hernia defect width was 9.97 ± 2.97 cm, with an equal proportion of W2 and W3 hernias. Twelve (27.3%) had loss of domain. The CSI was >0.146 in 52.3% of the hernias. The RDR was inferior to 1.34 in the same percentage of patients.

**TABLE 1:** Baseline and hernia characteristics.

Variable	N (%)
Age, years (mean)	63.3 ± 10.3
Sex	25 (56.8%) male; 19 (43.2%) female
BMI, kg/m^2^ (mean)	28.9 ± 4.3
Smoking/COPD	9 (20.5%)
Anticoagulation	7 (15.9%)
Diabetes	12 (27.3%)
ASA classification	
ASA II	30 (68.2%)
ASA III	13 (29.5%)
ASA IV	1 (2.3%)
Recurrent hernia	20 (46.5%)^a^
Previous mesh	14 (38.8%)^b^
Defect width, cm (mean)	9.97 ± 2.97
EHS classification (M)	
M1-3	4 (9.1%)
M1-4	3 (6.8%)
M1-5	1 (2.3%)
M2	1 (2.3%)
M2-3	8 (18.2%)
M2-4	12 (27.3%)
M2-5	1 (2.3%)
M3	8 (18.2%)
M3-4	3 (6.8%)
M3-5	2 (4.5%)
M5	1 (2.3%)
EHS classification (W)	
W2	22 (50%)
W3	22 (50%)
m-VHWGS	
Grade 1	9 (20.5%)
Grade 2	31 (70.5%)
Grade 3	4 (9.1%)
Loss of Domain	12 (27.3%)
Rectus/Defect ratio	
RDR <1.34	23 (52.3%)
Component Separation Index	
CSI >0.146	23 (52.3%)
Retro-rectus space, cm (mean)	12.95 ± 3.09

^a^ 1 missing value.

^b^ 8 missing values.

Eleven patients (25%) were enrolled in a surgical and/or non-surgical weight loss program before surgery. In this subgroup of patients, the mean initial and preoperative BMI were 39.8 ± 5.2 kg/m^2^ and 30.0 ± 5.6 kg/m^2^, respectively. Bariatric surgery was performed on eight patients, most of them before the abdominal wall reconstruction.

In 61.4% of the surgeries, the abdominal wall reconstruction was performed without CST. When it was needed, TAR was most frequently used (Table 2). The rate of posterior and anterior fascial closure was 100%. Ninety-six percent of the cases underwent adjuvant BTA. The median time from BTA to surgery was 38 days (31–55). PPP was performed in 25% of patients and IFT in 20.5%. There was no morbidity associated with BTA. However, two complications occurred during the PPP, one patient developed atrial fibrillation with rapid ventricular response and there was an inadvertent placement of catheter into subcutaneous tissue.

**TABLE 2:** Operative data.

Variable	N (%)
Surgical technique	
Rives-Stoppa	27 (61.4%)
Unilateral TAR	6 (13.6%)
Bilateral TAR	9 (20.5%)
Bilateral ACS	1 (2.3%)
Unilateral TAR + Unilateral ACS	1 (2.3%)
Other procedures	
Panniculectomy	12 (27.3%)
Vertical Gastrectomy	1 (2.3%)
2nd stage Duodenal Switch	1 (2.3%)
Operative time, min (mean)	172.8 ± 43.7
Mesh dimensions	
Width, cm (median)	15 (12–19)
Length, cm (median)	30 (27–30)
ICU admission	21 (47.7%)

In the postoperative period, early complication rate was 20.5% (Table 3). Most of the complications were surgical site occurrences and classified with a Clavien Dindo I-IIIA [21]. The rate of superficial surgical site infection, seroma, and haemorrhagic complications was 6.8% for each complication among the patients. Additionally, 4.5% of patients experienced skin and subcutaneous dehiscence. One patient required surgical reintervention 20 months after the initial surgery due to an interparietal hernia, and a TAR was performed. Another case of hernia recurrence was reported 13 months after a Rives-Stoppa. These patients were submited to BTA and BTA + IFT, respectively. During a median follow-up of 13 months (8–25), late complications occurred in four patients. Two patients with mesh infection underwent surgical removal of the infected mesh, 11 and 24 months after the initial surgery. Both patients had significant comorbidities, including obesity and diabetes, and underwent BTA plus PPP followed by TAR.

**TABLE 3:** Postoperative complications and follow up.

Variable	N (%)
Intraoperative complications	5 (11,4%)
Intestinal perforation	5 (11.4%)
Diaphragm injury	1 (2.3%)
Early complications (<30 days)	9 (20.5%)
Seroma	3 (6.8%)
Wound dehiscence (skin and subcutaneous tissue)	2 (4.5%)
Superficial surgical site infection	3 (6.8%)
Hematoma	1 (2.3%)
Arterial active bleeding	2 (4.5%)
Respiratory infection	1 (2.3%)
Interparietal hernia	1 (2.3%)
Late complications (>30 days)	4 (9.1%)
Seroma	2 (4.5%)
Mesh infection	2 (4.5%)
Recurrence	2 (4.5%)
Duration of follow-up, months (median)	13 (8–25)

Comparing the abdominal wall reconstruction surgeries with and without CST, no significant statistical differences were observed in hernia characteristics, adjuvant treatments, or postoperative outcomes. However, a trend was observed towards a higher proportion of M1 hernias (23.5% vs. 14.8%) and hernias with loss of domain (41.2% vs. 18.5%) in the CST group. The opposite was true for early complications rate (11.8% vs. 25.9%). Operative time and mesh width were higher in the CST group. The width of the hernia defect and the retrorectus space did not differ between the two groups. In patients with defects larger than 8 cm in width, 60% underwent repair without CST (Table 4).

**TABLE 4:** Comparing group without CST versus group with CST.

Variable	Without CST	CST	p-value
Number	27	17	
Demographics			
Age, years (mean)	65.0 ± 10.7	60.71 ± 9.3	p = 0.180
BMI, kg/m^2^ (mean)	28.5 ± 4.6	29.4 ± 3.8	p = 0.504
Smoking/COPD	5 (18.5%)	4 (23.5%)	p = 0.716
Characteristics of hernia			
Defect, cm (mean)	9.74 ± 2.69	10.34 ± 3.43	p = 0.525
Defect >8 cm	20 (74.1%)	13 (76.5%)	p = 1.000
W3	13 (48.1%)	9 (52.9%)	p = 0.757
M1 region	4 (14.8%)	4 (23.5%)	p = 0.690
M5 region	3 (11.1%)	2 (11.8%)	p = 1.000
Retro-rectus space, cm (mean)	12.95 ± 3.34	12.60 ± 3.02	p = 0.729
Loss of Domain	5 (18.5%)	7 (41.2%)	p = 0.164
RDR <1.34	14 (51.9%)	9 (52.9%)	p = 0.944
CSI >0.146	15 (55.6%)	8 (47.1%)	p = 0.583
Adjuvants			
BTA	25 (92.6%)	17 (100%)	p = 0.515
PPP	6 (22.2%)	5 (29.4%)	p = 0.724
IFT	6 (23.1%)	3 (17.6%)	p = 1.000
Surgery			
Operative time, min (mean)	153.2 ± 28.4	221.8 ± 36.4	**p = 0.002**
Mesh width, cm (median)	14.5 (11.5–15)	18.5 (15–26.5)	**p = 0.027**
Mesh length, cm (median)	30 (24–30)	30 (29–33.5)	p = 0.121
Postoperative			
ICU admission	12 (44.4%)	9 (52.9%)	p = 0.583
Total length of stay, days (mean)	5.3 ± 3.3	5.8 ± 1.7	p = 0.572
Early complications	7 (25.9%)	2 (11.8%)	p = 0.445
Late complications	1 (4.3%)	3 (20%)	p = 0.282
Recurrence	1 (3.7%)	1 (5.9%)	p = 1.000

p < 0.05 values indicated in bold.

A subset analysis was performed in hernias with CSI >0.146 and RDR <1.34. In patients with CSI >0.146, 65.2% underwent abdominal wall reconstruction without CST. Of those patients, 60% were W3 hernias and three patients had loss of domain. In the RDR <1.34 group, 60.9% did not require CST for abdominal wall closure. 58.8% were W3 hernias and two patients had loss of domain.

## Discussion

In our study, 61.4% of patients underwent surgery without CST after the use of adjuvant therapies, a percentage higher than previously reported by others [10, 20, 26]. The impact of adjuvant therapy on abdominal wall musculature before complex hernia surgery has been demonstrated in previous studies [10] however, information regarding their effect on final surgical technique remains limited. The available evidence primarily originates from systematic reviews and retrospective studies [8, 27–29].

The overall complication rate was 20.5% and 9.1% in the early and late postoperative period, respectively. These rates are lower than those reported in other studies [7, 26, 30]. Factors that may have contributed to these outcomes include a strong emphasis on the preoperative rehabilitation of patients, particularly concerning weight loss. The average BMI in our study was lower than that reported in other investigations [30, 31]. In contrast to previous studies, the complication rate in patients undergoing CST was not found to be higher than that in patients who did not undergo this procedure [24, 31].

Concerning the recurrence rate, two cases were observed during follow-up: one following a Rives-Stoppa repair, complicated by postoperative retrorectus bleeding, and another after a TAR, presenting with an intraparietal hernia. Nonetheless, our recurrence rate is lower than that reported in the existing literature [20, 28]. However, the median follow-up of 13 months may influence the findings, limiting the extrapolation of medium- and long-term conclusions.

There is established evidence of the reshaping of the abdominal cavity after adjuvant therapy with BTA. When applied at the lateral abdominal wall musculature, results in their elongation [32]. The increased length and reduced thickness of the lateral muscles, as well as the decrease in the width of the hernia defect, increase abdominal wall compliance [10, 29]. Due to the demonstrated benefits within our group, over recent years, the cutoff for utilization has progressively decreased and is now applied to hernias larger than 8 cm. Nonetheless, adjuvant therapies may prove beneficial in selected cases of smaller hernias. Careful, individualized assessment remains essential, as certain patients may require therapeutic strategies beyond those defined in the current algorithm. Tailoring treatment to specific patient or hernia-related factors may ultimately enhance clinical outcomes in appropriately selected cases.

Midline closure seems to be more related to compliance due to the elasticity of the muscle fibres elicited by the BTA blockade, rather than solely due to the value found for lateral fibre advancement [26]. Whether this effect is responsible for the downsizing of the hernia and prevention of the need for CST in some cases remains to be determined. Although the maximum effects of muscle paralysis is achieved after 3–4 weeks, its effect lasts for 6–9 months, allowing the abdominal wall to heal in a low-tension environment [33]. It is not yet known whether, regardless of the technique used, achieving midline closure with less tension is associated with a lower recurrence rate [29].

PPP induces a mechanical stretching of muscle fibres, that combined with the relaxation induced by BTA, increases the compliance of the abdominal wall and the volume that can be accommodated within the abdominal cavity. This combined approach of techniques was successfully used in a quarter of our patients. The gradual and steady increase in intra-abdominal pressure improves respiratory function adaptation and decreases the likelihood of developing a quaternary compartment syndrome in the postoperative period [13]. Because of that, in our group, those hernias presenting with a tight defect characterized by a “mushroom” pattern are assigned for adjuvant treatment with PPP, an indication never reported before to our knowledge.

In hernias with a greater separation between the rectus muscles and significant myofascial retraction of the lateral abdominal muscles, particularly in the middle and lower abdomen, better outcomes can be achieved with BTA muscle blockade combined with IFT. In these cases, PPP may result in insufflation of the hernia sac, causing stretching of the skin and subcutaneous tissue rather than increasing the abdominal cavity volume through lateral muscle elongation. This time-limited traction, applied during surgery, can facilitate a tension-free closure of the abdominal wall by stretching the temporarily paralyzed muscles [9].

In our study, 45.5% of patients received a combination of two adjuvant techniques due to their presumed synergy in hernia downsizing. Rather than applying all adjuvant methods simultaneously, we utilize a tailored approach based on specific clinical criteria. Our strategy does not prioritize one adjuvant over another; instead, we acknowledge that each technique has distinct roles and is best suited for each clinical scenario. The comprehensive availability and expertise in utilizing these adjuvant methods contribute to enhanced patient care and allow for the individualized tailoring of treatment strategies.

The benefits of using adjuvants do not come without limitations. Although economic costs were not addressed in this study, the expenses associated with BTA and IFT are considerable, and PPP entails prolonged hospital stays with inherent costs. Outpatient protocols are already being described [34]. Regarding morbidity, we observed two cases of complications during the PPP, while other studies stated that complications can be as high as 25% [8].

The width of the hernia defect is considered a criterion that can define the need for CST. Traditionally, the Rives-Stoppa technique is an option for hernias up to 10 cm and thus possibly not applicable to W3 hernias [35]. In our study, 59.1% of patients with W3 hernias achieved abdominal wall closure without CST. Additionally, the width of the hernia defect did not differ between the two groups, showing the importance of factors other than diameter in determining hernia complexity.

Regarding hernia location, despite a higher proportion of patients with M1 hernias in the CST group, our study found no significant differences in the rate of component separation based on the hernia site, in contrast to the findings of some other authors [31]. There is still no evidence whether CST after adjuvant therapy can be reduced in certain hernia groups.

Another point of discussion is the size of the mesh used and its impact on the recurrence rate. The width, and consequently the overlap of the mesh, was smaller in the group of patients where CST was avoided. Whether downstaging the hernia with reduced mesh overlap will adversely affect long-term outcomes remains to be determined, and we aim to address this question through prospective follow-up of these patients.

No factors related to the patient or hernia characteristics were identified as predictors of the surgical technique used. Even in the subgroup of patients for whom CSI and RDR predicted the need for CST, it was avoided in 65.2% and 60.9% of the cases, respectively. This demonstrates the limitation of applying these tools to patients undergoing adjuvant treatments for hernia downstage.

Our study has several limitations that must be acknowledged. First, its retrospective design inherently restricts the depth of data that could be collected. Additionally, the absence of a control group, as we did not include all other hernia cases that were not treated with adjuvants, precludes the comparative analysis. Furthermore, we did not perform follow-up CT scans after adjuvant therapy, leaving unanswered whether certain subgroups of patients may experience a more pronounced benefit from these therapies. It did not alter our surgical approach, given that we initiated with a Rives-Stoppa technique and performed TAR on demand. It is also important to consider that, assessment of tension during closure is subjective, and some variability between surgeons may had occurred. The strict application of our inclusion criteria to the patient cohort may have resulted in the exclusion of certain patients with smaller hernias who were not included in our study but who may nonetheless require component separation. We included two cases in which bariatric surgery was performed simultaneously with abdominal wall reconstruction, as preoperative bariatric surgery was not feasible. We acknowledge that this may introduce bias.

However, several strengths can be mentioned. To the best of our knowledge, this study is the first to demonstrate the simultaneous effects of different adjuvant therapies employed both before and during surgery, an effect that we believe is amplified by their combined application. Despite the small number of patients in our cohort, we are not aware of any previous studies reporting a comparable sample size. Furthermore, our focus on preoperative rehabilitation—particularly in managing patients with obesity—and the establishment of criteria for the use of BTA, PPP, and IFT contribute to reducing treatment heterogeneity.

Despite the encouraging results, individual responses to adjuvant therapies may vary among patients. The selection of patients or hernias that would benefit the most from each approach is still unknown, and further research is needed. Maintaining a holistic and individualized approach for each patient is critical in hernia repair.

## Conclusion

The use of adjuvant therapies in patients with complex ventral hernias is safe and may influence the surgical strategy for abdominal wall reconstruction, potentially influencing the use of a less disruptive surgical technique. A structured approach to their application, guided by specific clinical criteria, may enhance their effectiveness and provide additional benefits.

## Data Availability

The original contributions presented in the study are included in the article/supplementary material, further inquiries can be directed to the corresponding author.
